# Quantitative Comparison of the Marker Compounds in Different Medicinal Parts of *Morus alba* L. Using High-Performance Liquid Chromatography-Diode Array Detector with Chemometric Analysis

**DOI:** 10.3390/molecules25235592

**Published:** 2020-11-27

**Authors:** Jung-Hoon Kim, Eui-Jeong Doh, Guemsan Lee

**Affiliations:** 1 Division of Pharmacology, School of Korean Medicine, Pusan National University, Yangsan 50612, Korea; kmsct@pusan.ac.kr; 2 Research Center of Traditional Korean Medicine, Wonkwang University, Iksan 54538, Korea; bluemoon-lion@hanmail.net; 3 Department of Herbology, College of Korean Medicine, Wonkwang University, Iksan 54538, Korea

**Keywords:** *Morus alba* L., medicinal parts, chemical compounds, quantitative analysis, chemometric analysis

## Abstract

It is thought that the therapeutic efficacy of *Morus alba* L. is determined by its biological compounds. We investigated the chemical differences in the medicinal parts of *M. alba* by analyzing a total of 57 samples (15 root barks, 11 twigs, 12 fruits, and 19 leaves). Twelve marker compounds, including seven flavonoids, two stilbenoids, two phenolic acids, and a coumarin, were quantitatively analyzed using a high-performance liquid chromatography-diode array detector and chemometric analyses (principal component and heatmap analysis). The results demonstrated that the levels and compositions of the marker compounds varied in each medicinal part. The leaves contained higher levels of six compounds, the root barks contained higher levels of four compounds, and the twigs contained higher levels of two compounds. The results of chemometric analysis showed clustering of the samples according to the medicinal part, with the marker compounds strongly associated with each part: mulberroside A, taxifolin, kuwanon G, and morusin for the root barks; 4-hydroxycinnamic acid and oxyresveratrol for the twigs and skimmin; chlorogenic acid, rutin, isoquercitrin, astragalin, and quercitrin for the leaves. Our approach plays a fundamental role in the quality evaluation and further understanding of biological actions of herbal medicines derived from various medicinal plant parts.

## 1. Introduction

*Morus alba* L. belongs to the Moraceae family. It is a deciduous shrub or tree that is grown naturally, and is widely cultivated for medicinal and edible purposes. Four *M. alba* parts have been used medicinally, as registered in the Korean pharmacopeias, including the twigs (Sang-Ji, Mori Ramulus), leaves (Sang-Yeop, Mori Folium), fruits (Sang-Sim-Ja, Mori Fructus), and root barks (Sanb-Baek-Pi, Mori Radicis Cortex), each of which have different therapeutic efficacies [1,2]. Each medicinal *M. alba* part has various pharmacological effects due to its specific phytochemicals. For example, twigs have hepatoprotective effects from stilbenes [3], root barks have phosphodiesterase-4 inhibitory effects from flavonoids [4], fruits have anti-diabetic activities from phenolic compounds and flavonoids [5], and leaves have anti-cancer effects from phenolic compounds [6]. Furthermore, each medicinal part presents different chemical compositions, and shows distinguishable pharmacological effects, such as the differences in the anti-oxidant, anti-microbial, anti-diabetic, anti-inflammatory, and anti-tyrosinase effects of the leaves, fruits, twigs, stems, and root barks [7,8,9].

Phytochemical content is usually specific to each plant organ. For example, *Empetrum hermaphroditum* contains abundant anthocyanins and flavanols in the fruits, stilbenoids in the leaves, and catechins, procyanidins, and proanthocyanidins in the stems and roots [10]. *Lycium intricatum* has different phenolic compound and flavonoid levels in the fruits and leaves [11]. These properties are due to the fact that the genes in charge of phytochemical biosynthesis are differentially expressed in various plant organs and tissues. For example, gene expression differences in the different organs of *Camellia sinensis* results in biosynthesis differences in flavonoids, caffeine, and theanine [12]. Similarly, differences in cytochrome P450 genes are responsible for differences in diterpene biosynthesis in the different organs of *Coffea arabica* [13]. Organ-specific differences in phytochemicals allows for each organ to fulfil its function, such as plant growth, herbivore, and microbial defense, pollinator attraction, flowering, pigment development, fruit development, and ripening, or allelopathy [13,14,15,16]. Hence, determining the phytochemical contents of each plant part is an initial and crucial research step, since these chemical differences might contribute to variations in the biological or pharmacological efficacies of each part.

Analytical tools, including high-performance liquid chromatography (HPLC) or liquid chromatography/mass spectrometry (LC/MS), coupled with chemometric analyses (i.e., principal component analysis, hierarchical clustering analysis, and discriminant analysis) have been recognized as comprehensive and useful techniques for the discrimination of geographical plants origins, chemotaxonomic authentication of botanical species, and quality evaluations of herbal medicines [17,18,19]. These tools also make it possible to differentiate the chemical characteristics of plants by parts, by identifying the phytochemicals strongly associated with such differentiations [20,21,22]. Thus, it is highly applicable to investigate the organ-specific phytochemicals in medicinal plants with two more therapeutic parts that are readily available, as is the case with *M. alba*.

Previous studies compared the chemical compositions of different *M. alba* parts using HPLC analysis [9,23] with orthogonal projection for latent structures discriminant analysis [23]; however, the sample size for each medicinal part was insufficient, and the statistical comparison did not investigate quantitative differences in the marker compounds of the medicinal parts. In this study, a simultaneous analytical method was established and validated that determine 12 marker compounds in the four medicinal *M. alba* parts. The quantitative differences in these marker compounds were compared statistically, and chemometric analysis (i.e., principal components analysis and heatmap analysis) was used to investigate the chemical characteristics of each medicinal part and their related compounds.

## 2. Results

### 2.1. Optimization of the Sample Extraction and the Analytical Conditions

For better extraction efficiencies of the 12 marker compounds, ultrasonic extraction of the Morus samples was conducted at multiple solvent ratios (methanol:water = 10:0, 7:3, and 5:5). The 70% methanol mixture showed balanced extraction efficiencies of the 12 marker compounds (i.e., the middle values of the compounds’ absolute peak areas), compared with the methanol alone and the 50% solvent mixture; therefore, the Morus samples were extracted using the 70% methanol mixture for 30 min with the ultrasonic extractor.

**Figure 1:**
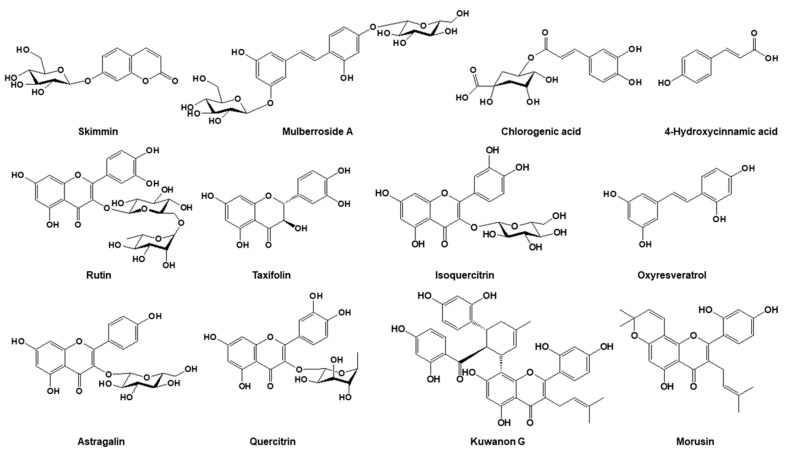
Chemical structures of the 12 marker compounds from the Morus samples.

The HPLC analytical conditions were optimized by adjusting the mobile phase modifier, the mobile phase composition, and the UV detection wavelength. The aqueous mobile phase composition with acetonitrile was compared to water alone and water with modifiers, including 0.1% Formic acid (FA) (*v/v*) and 0.1% Trifluoroacetic acid (TFA) (*v/v*). The mobile phase composition of 0.1% TFA containing water and acetonitrile showed higher peak presence and better interpeak separations of the marker compounds. The UV wavelengths for the diode array detector were selected using the optimal UV absorbance for each peak: UV 255 nm for rutin, isoquercitrin and quercitrin; UV 265 nm for astragalin and kuwanon G; UV 275 nm for morusin; UV 290 nm for taxifolin; UV 310 nm for 4-hydroxycinnamic acid; and UV 325 nm for skimming, mulberroside A, chlorogenic acid, and oxyresveratrol (Figure 1 and Table 1).

**Figure 2:**
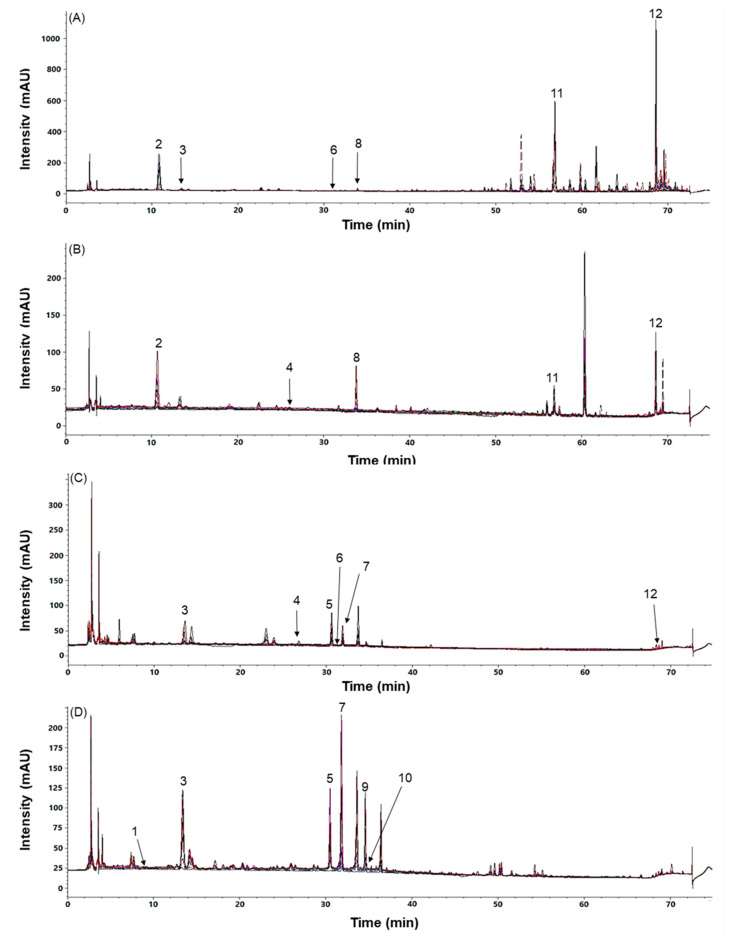
Overlapped chromatograms of the representative samples of the root barks (**A**), the twigs (**B**), the fruits (**C**), and the leaves (**D**) of the Morus samples at the detection wavelength of 265 nm; 1, skimmin; 2, mulberroside A; 3, chlorogenic acid; 4, 4-hydroxycinnamic acid; 5, rutin; 6, taxifolin; 7, isoquercitrin; 8, oxyresveratrol; 9, astragalin; 10, quercitrin; 11, kuwanon G; 12, morusin.

The interday precision of the marker compounds were <5.0% (relative standard deviation, RSD value) with accuracies of 90.46–104.65%. The interday precisions were <8.7% (RSD value) with accuracies of 87.74–103.67% (Table S1). The recoveries of the marker compounds ranged from 84.06% to 115.30% with RSD value values of <8.6% (Table S2). The above-developed method was successfully applied to analyze the 12 marker compounds (Figure 2 and Figure S1).

### 2.2. Quantitative Comparison of the 12 Marker Compounds in Different Medicinal Parts of M. alba

**Figure 3:**
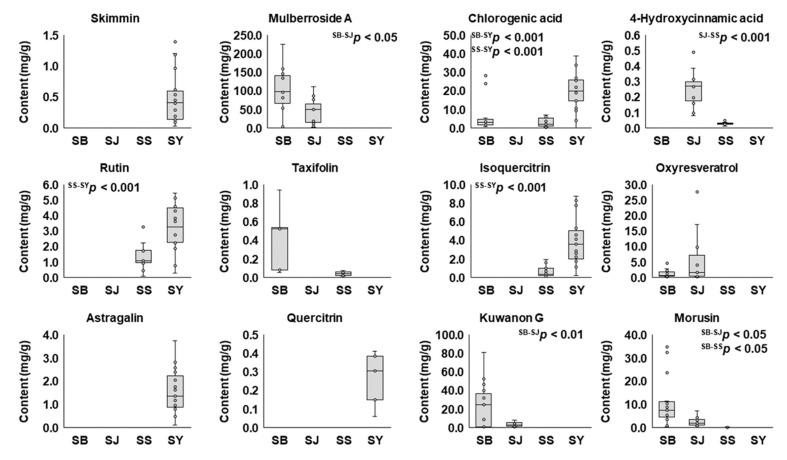
The contents of the 12 marker compounds in different medicinal parts of the Morus samples. SB, root barks; SJ, twigs; SS, fruits; SY, leaves. The statistically significant differences between the medicinal parts are represented at *p* < 0.05, *p* < 0.01 or *p* < 0.001.

The contents of the 12 marker compounds varied considerably in the samples from single medicinal parts, and between the different medicinal parts (Table S3). Significantly higher contents of mulberroside A, kuwanon G, and morusin were contained in the root barks (100.19 ± 63.62 mg/g, 24.05 ± 23.17 mg/g, and 10.98 ± 10.49 mg/g, respectively) than in the twigs (44.55 ± 34.61 mg/g, 3.86 ± 2.54 mg/g, and 2.63 ± 1.97 mg/g). Morusin was also found in significantly low amounts in the fruit samples (0.11 ± 0.10 mg/g) compared to the root barks. In contrast, oxyresveratrol showed higher amounts in the twigs (6.15 ± 8.47 mg/g) than in the root barks (1.27 ± 1.19 mg/g), but the difference was not statistically significant. The contents of chlorogenic acid, rutin, and isoquercitrin were significantly greater in the leaves (19.99 ± 9.36 mg/g, 3.25 ± 1.47 mg/g, and 3.83 ± 2.42 mg/g, respectively) than in the fruits (3.21 ± 2.40 mg/g, 1.35 ± 0.84 mg/g, and 0.66 ± 0.60 mg/g, respectively) and the root barks (6.77 ± 8.81 mg/g for chlorogenic acid). The 4-hydroxycinnamic acid content was significantly higher in the twigs (0.26 ± 0.11 mg/g). Taxifolin was non-significantly higher in the root barks (0.43 ± 0.33 mg/g). Both of these were higher than in the fruits (0.03 ± 0.01 mg/g for 4-hydroxycinnamic acid and 0.04 ± 0.02 mg/g for taxifolin). Skimmin and astragalin were detected only in the leaves of *M. alba* (0.49 ± 0.41 mg/g and 1.55 ± 0.93 mg/g, respectively). Quercitrin was only found in small amounts in some of the leaf samples (5 out of 19 samples), with a content of 0.26 ± 0.14 mg/g (Figure 3).

### 2.3. Chemometric Analysis of the Marker Compounds in the Morus Samples

**Figure 4:**
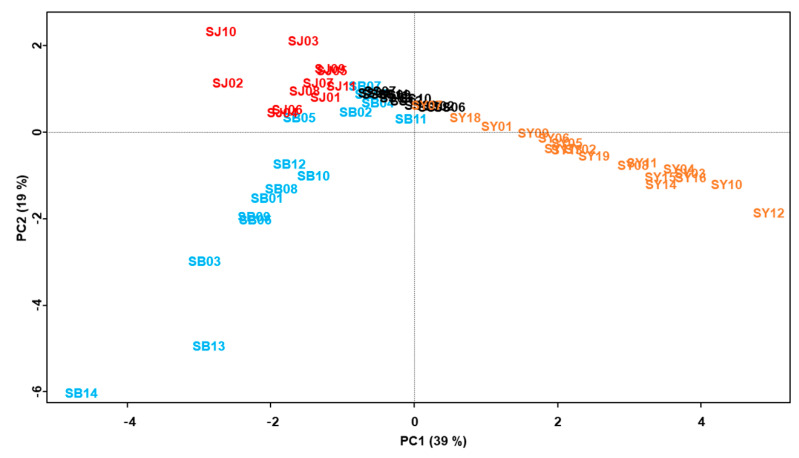
The principal component score plot (PC1 vs. PC2) from the 12 marker compound contents of the Morus samples. PC1 and PC2 contributed to 39% and 19% of the total variance, respectively. SB, root barks; SJ, twigs; SS, fruits; SY, leaves.

In the principal component (PC) score plot, samples from single medicinal parts had similar PC1 and PC2 scores. The root bark samples (SBs) had negative PC1 and PC2 scores, except for SB02, −04, −05, −07, −11, and −15. The positive SBs overlapped with the positive PC2 scores of the fruit samples. The twig samples (SJs) were negative for PC1 and positive for PC2. The fruit samples (SSs) were in the narrow ranges of positive and negative PC1 scores, and positive PC2 scores. The leaf samples (SYs) were positive for PC1 and negative for PC2, except for SY01, −07, and −18. The positive SYs also overlapped with the fruit samples for positive PC2 scores (Figure 4).

**Figure 5:**
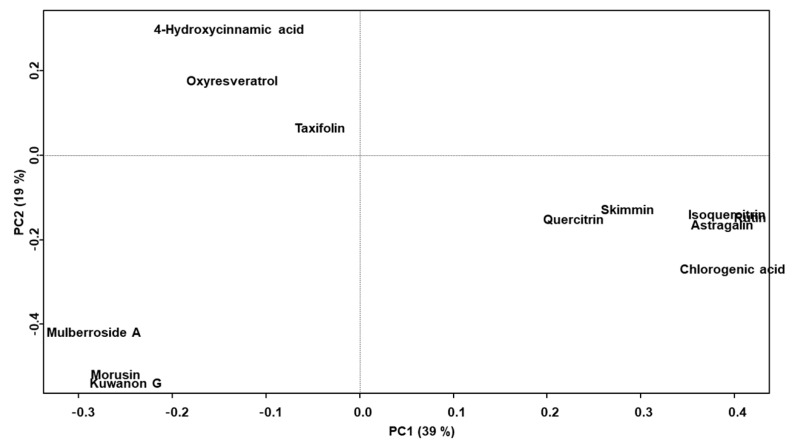
The principal component loading plot (PC1 vs. PC2) from the 12 marker compound contents of the Morus samples. PC1 and PC2 contributed to 39% and 19% of the total variance, respectively.

The distribution of the 12 marker compounds in the PC loading plot showed PC scores relevant to the medicinal parts in which they were contained: skimmin, chlorogenic acid, rutin, isoquercitrin, astragalin, and quercitrin had positive PC1 and negative PC2 scores; mulberroside A, kuwanon G and morusin had negative PC1 and PC2 scores, and 4-hydroxycinnamic acid, oxyresveratrol and taxifolin had negative PC1 and positive PC2 scores (Figure 5).

**Figure 6:**
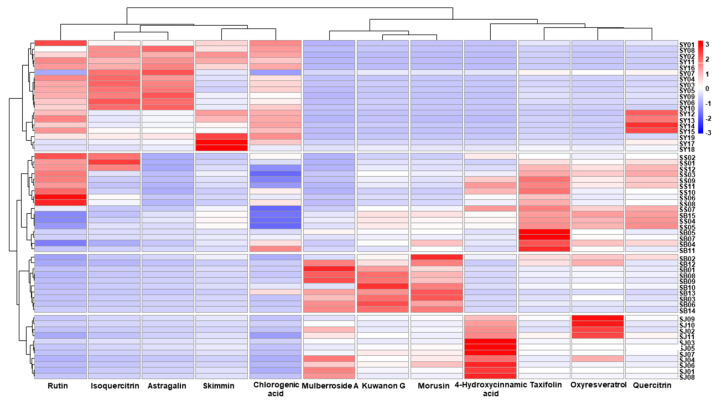
Clustered heatmap of the Morus samples and the marker compounds. SB, root barks; SJ, twigs; SS, fruits; SY, leaves.

These relationships were marked as a dip red color and were observed more distinctively in the clustered heatmap. The Morus samples were obviously divided into four groups according to medicinal part, except for SB04, −05, −07, −11, and −15, which were clustered in the fruit samples. Rutin, isoquercitrin, astragalin, skimmin, and chlorogenic acid were mainly prominent in the leaf samples, while rutin and isoquercitrin were also apparent in some fruit samples. Mulberroside A, taxifolin, kuwanon G, and morusin were most obvious in the root bark samples, but lesser so in the twig samples. Moreover, 4-Hydroxycinnamic acid and oxyresveratrol were apparent in the twig samples. Quercitrin was found in some of the leaf samples, and its relation to other compounds was unlike the distribution of the PC loading plot (Figure 6).

## 3. Discussion

The phytochemical levels, particularly the secondary metabolites, showed part-specific or tissue-specific variation of single plants, which corresponds to the fact that gene expression for phytochemical biosynthesis is highly plant-part dependent [24,25,26]. However, some compounds represented significant variations of the contents even in same medicinal part, presumably due to intra-species factors (i.e., different levels of the secondary metabolite biosynthesis, deposit or transformation) or extrinsic factors (i.e., circumstance of growing, harvesting season, degree of sample dryness, etc.).

In our findings, chlorogenic acid (3-caffeoylquinic acid) was found at the highest amount in *M. alba* leaves, followed by rutin (quercetin-3-O-rutinoside) and isoquercitrin (quercetin-3-O-glucoside), as previously reported [27,28]. Chlorogenic acid is preferentially formed in the leaves of *M. alba* by shikimate/quinate hydroxycinnamoyltransferase (HCT), particularly MaHCT4, during the chlorogenic acid biosynthetic process [29]. Isoquercitrin is derived from quercetin primarily in *M. alba* leaves via flavonol-3-O-glucosyltransferase (F3GT), and is subsequently transformed to rutin via flavonol-3-O-glucoside-L-rhamnosyltransferase (UGT78D1), particularly MaUGT78D1, which was expressed more in the leaves than in the other medicinal parts during the glycosylation of flavanols [30,31]. Moreover, skimmin (umbelliferone-glucoside) and astragalin (kaempferol-3-O-glucoside), which were found only in the leaves, were also considered to be distinct compounds in *M. alba* leaves [28,32,33], where F3GT catalyses the biosynthesis of astragalin [31]. Quercitrin was found in a wide variation in the leaves of the different leaf samples, but its presence was not supported by previous studies.

Higher levels of mulberroside A (oxyresveratrol diglucoside) in the root bark is also confirmed by a previous study [34]. The root is considered a substantial site for the biosynthesis of mulberroside A via glycosylation of oxyresveratrol (hydroxy-resveratrol), which is found as a precursor in the root of *M. alba* [35]. Oxyresveratrol is known as one of the key compounds in *M. alba* twigs, following its mono- or di-glucosylated moieties [36,37,38], which supports the non-statistically significant higher amount in the twigs than in the root barks in this study.

It is reported that kuwanon G, an isoprenylated-flavone derivate, is synthesized and found in the root barks of *M. alba* as a Diels-Alder adduct of a chalcone and a dehydroprenylflavone [39,40,41,42]. Another isoprenylated flavone, morusin, is also contained primarily in the root barks of *M. alba* [43,44]. A previous study reported that chalcone flavanone isomerase and flavonoid 3,5-hydroxylase, which are found only in roots, participate in flavonoid biosynthesis, which explains the characteristically higher accumulation of the two flavones in the root of *M. alba* [45]. Two root bark samples (SB7 and SB11) did not contained mulberroside A, but contained taxifolin (dihydroquercetin) and interestingly very lower levels of kuwanon G and morusin. It is assumed that unexplained intrinsic or extrinsic factors disrupt the biosynthesis of mulberroside A, kuwanon G, and morusin simultaneously in two SB samples above.

Previous studies report that 4-hydroxycinnamic acid (*p*-coumaric acid) is derived from cinnamic acid via the P450-dependent enzyme 4-cinnamic acid hydroxylase [46]. It has been primarily found in the fruits of *M. alba* at lower levels than other compounds [47,48], but this study indicates that the twigs of *M. alba* may be another key accumulation part for 4-hydroxycinnamic acid, as previously reported [49].

The relation between plant part and marker compound was investigated using chemometric analyses, including principal component analysis and heatmap analysis. The PC score plot demonstrated that the *M. alba* samples showed confirmable distinguishment from other parts by their PC1 and PC2 scores (responsible for 58% of total variance), except for some overlapped samples of the root barks (SB) and leaves (SY). The PC scores of the loading plot explain the influential compounds in the clustering of the samples [50]: the root bark samples (SBs) were clustered mainly by mulberroside A, taxifolin, kuwanon G and morusin; the twig samples (SJs) were clustered mainly by 4-hydroxycinnamic acid and oxyresveratrol; and the leaf samples (SYs) were clustered mainly by skimmin, chlorogenic acid, rutin, isoquercitrin, astragalin and quercitrin. Likewise, the clustered heatmap, which visualizes the relationships between clusters and influential markers, shows that the *Morus* samples were distinguished into four distinctive clusters, according to their medicinal parts and their highly associated marker compounds. This result is similar to the PC score and loading plot results, except for quercitrin.

## 4. Materials and Methods 

### 4.1. Chemicals and Reagents

Analytical-grade acetonitrile, methanol, and water were purchased from J.T. Baker Inc. (Phillipsburg, NJ, USA). Formic acid (FA) was purchased from Fisher Scientific International Inc. (Pittsburgh, PA, USA). Trifluoroacetic acid (TFA) was purchased from Sigma-Aldrich (St Louis, MO, USA). Skimmin, chlorogenic acid, 4-hydroxycinnamic acid, rutin, taxifolin, isoquercitrin, oxyresveratrol, astragalin, quercitrin, kuwanon G and morusin were purchased from ChemFace (Wuhan, Hubei, China). The chemical structures of the marker compounds are shown in Figure 1.

*M. alba* root barks (SB), twigs (SJ), fruits (SS) and leaves (SY) (15 root bark samples, 11 twig samples, 12 fruit samples, and 19 leaf samples) were collected from the habitats in Korea, purchased from the herbal company (Kwangmyungdang; Ulsan, Korea), or were provided by the Korea Institute of Oriental Medicine (Naju, Jeonnam, Korea) (Table S4). The samples were authenticated by the authors (J. H. Kim and G. Lee). Voucher specimens (2020-PNUKM-SB01–SB15, SJ01–SJ11, SS01–SS12, and SY01–SY19) have been deposited at the School of Korean Medicine, at the Pusan National University (Yangsan, Gyeongnam, Korea).

### 4.2. Sample Preparation

Dried *M. alba* samples were pulverized and homogenized using a 500 μm testing sieve (Chunggyesanggong-sa; Gunpo, Gyeonggi, Korea). The powder (40 mg) was weighed and extracted with 1 mL of 70% methanol (methanol:water = 7:3, *v/v*) for 30 min using an ultrasonic extractor (Power Sonic 520; Hwashin Tech, Daegu, Korea). The extracted solution was centrifuged at 10,000 rpm for 2 min, then the supernatant was transferred to a 1.5 mL polypropylene tube after filtration through a 0.2 μm syringe filter (BioFact, Daejeon, Korea). The filtered extract was evaporated using a nitrogen-blowing concentrator (MGS2200; Eyela, Miyagi, Japan). The concentrated extract was dissolved in an HPLC-grade solvent mixture (methanol:water = 7:3, *v/v*) prior to HPLC injection.

### 4.3. HPLC Analytical Conditions

The quantitative analysis of the marker compounds was performed using an Agilent 1260 liquid chromatography system (Agilent Technologies, Palo Alto, CA, USA) equipped with an autosampler, degasser, quaternary solvent pump, and diode array detector. Data was processed using ChemStation software (Agilent Technologies Inc., USA). Twelve marker compounds were separated on a Capcell Pak Mg II C_18_ column (4.6 mm × 250 mm, 5 μm; Shiseido, Tokyo, Japan) at 35 °C. The flow rate was set at 1 mL/min and an injection volume was set at 10 μL. The mobile phase consisted of water containing 0.1% TFA (solvent A) and acetonitrile (solvent B). A gradient elution was applied as follows: 10% (solvent B, %) for 0–5 min, 10–12% (solvent B, %) for 5–15 min, 12–20% (solvent B, %) for 15–27 min, 20–70% (solvent B, %) for 27––65 min, 70–95% (solvent B, %) for 65–66 min, held for 3 min, then re-equilibrated to 10% (solvent B, %) until the end of the analysis. The diode-array detector was set at ultraviolet wavelengths of 255, 265, 275, 290, 310, and 325 nm.

### 4.4. Validation of the HPLC Method

The compounds were dissolved in methanol at 1000 μg/mL to make stock solutions, then were serially diluted to produce working solutions for HPLC analysis for the construction of calibration curves. The linearity of the calibration curves was evaluated using correlation coefficients (*r*^2^). The limit of detection (LOD) and the limit of quantification (LOQ) were determined as signal-to-noise (S/N) ratios of 3 and 10, respectively.

The repeatability of the HPLC method was determined via a precision test by analyzing low, medium, and high concentrations of the above-mentioned solutions three times within one day (intraday precision) and over three consecutive days (interday precision). Precisions are given as relative standard deviations (RSDs) (where RSD (%) = [(standard deviation / mean) × 100]). 

The accuracy of the HPLC method was determined by testing the recoveries of low, medium, and high concentrations of the marker compounds added to the sample solutions. Recovery was calculated as follows: Recovery (%) = ((detected concentration − initial concentration)/spiked concentration) × 100.

### 4.5. Chemometric Statistical Analysis

The distributions of the *Morus* samples and their marker compounds were analyzed, then visualized using the chemometric tools. A matrix consisting of rows (*Morus* samples) and columns (marker compound contents) was used to perform the chemometric analyses. The quantitative differences in the 12 marker compounds were compared using the Tukey’s test. The differences were considered statistically significant at *p* < 0.05, *p* < 0.01 or *p* < 0.001. The Tukey’s test and chemometric analyses were performed using open-source software R (v. 4.0.2; The R Foundation for Statistical Computing).

## 5. Conclusions

The HPLC-DAD method was established, validated, and applied for the quantitative analysis of the 12 marker compounds in 57 different medicinal parts of *M. alba*, including 15 root bark samples, 11 twig samples, 12 fruit samples, and 19 leaf samples. The contents of the marker compounds varied by the medicinal part, with compound levels in the levels and root barks. Principal component analysis and heatmap analysis showed that the marker compounds were strongly associated in clusters for the different parts, such as mulberroside A, taxifolin, kuwanon G and morusin for the root barks; 4-hydroxycinnamic acid and oxyresveratrol for the twigs, and skimmin, chlorogenic acid, rutin, isoquercitrin, astragalin, and quercitrin for the leaves. The HPLC, coupled with chemometric analysis, provide a comprehensive understanding of the medicinal part-specific chemical markers in *M. alba*, a multisource-usable medicinal plant.

## Figures and Tables

**Table 1:** Regression equation, linear range, correlation coefficient (r^2^), limit of detection, and limit of quantification of the marker compounds.

Compound	*t_R_* (min)	UV (nm)	Regression Equation	Linear Range (μg/mL)	*r* ^2^	LOD (μg/mL)	LOQ (μg/mL)
Skimmin (**1**)	9.72	325	y = 7.885x + 3.792	1.88–30.00	0.9998	0.47	1.56
Mulberroside A (**2**)	10.70	325	y = 12.651x + 10.932	9.38–600.00	1.0000	0.59	1.95
Chlorogenic acid (**3**)	13.42	325	y = 26.686x + 43.967	10.94–700.00	0.9993	0.68	2.28
4-Hydroxycinnamic acid (**4**)	26.11	310	y = 86.121x + 0.582	0.31–10.00	1.0000	0.08	0.26
Rutin (**5**)	30.47	255	y = 17.363x + 23.435	4.38–280.00	0.9996	0.14	0.46
Taxifolin (**6**)	31.13	290	y = 31.091x + 3.466	0.63–20.00	0.9998	0.16	0.52
Isoquercitrin (**7**)	31.75	255	y = 23.558x − 7.984	4.69–150.00	1.0000	0.15	0.49
Oxyresveratrol (**8**)	33.80	325	y = 48.105x − 4.063	3.13–200.00	1.0000	0.10	0.33
Astragalin (**9**)	34.54	265	y = 26.060x − 1.994	1.56–100.00	1.0000	0.10	0.33
Quercitrin (**10**)	34.74	255	y = 29.297x + 2.644	0.78–50.00	0.9999	0.10	0.33
Kuwanon G (**11**)	56.83	265	y = 16.023x + 2.815	3.13–200.00	0.9999	0.20	0.65
Morusin (**12**)	68.62	275	y = 38.191x + 7.181	2.34–150.00	0.9999	0.07	0.24

*t_R_*, retention time; UV, detection wavelength; *r*^2^, correlation coefficient; LOD, limit of detection; LOQ, limit of quantification.

## Supplementary Materials

The following are available online, Figure S1: chromatograms of twelve marker compounds at the detection wavelength of UV 265 nm, Table S1: Intra- and interday precisions of the marker compounds, Table S2: Recoveries of the marker compounds (n = 3), Table S3: Mean contents (mg/g) of the marker compounds in the extracts of the *Morus* samples, Table S4: The samples of different medicinal parts of *Morus alba* L.
