# Supplementary material for: Quantitative Comparison of the Marker Compounds in Different Medicinal Parts of Morus alba L. Using High-Performance Liquid Chromatography-Diode Array Detector with Chemometric Analysis
Source: Molecules. 2020 Nov 27;25(23):5592. doi: 10.3390/molecules25235592 (PMC7730820; doi:10.3390/molecules25235592)
Supplement: Supplementary file 1 [file molecules-25-05592-s001.pdf]

Supplementary Materials

Quantitative Comparison of The Marker Compounds in Different Medicinal Parts of *Morus alba* L. Using High-Performance Liquid Chromatography-Diode Array Detector with Chemometric Analysis

Jung-Hoon Kim[1,†], Eui-Jeong Doh[2,†], Guemsan Lee[2,3,*]

1 Division of Pharmacology, School of Korean Medicine, Pusan National University, Yangsan 50612, Korea; kmsct@pusan.ac.kr
2 Research Center of Traditional Korean Medicine, Wonkwang University, Iksan 54538, Korea; bluemoon-lion@hanmail.net
3 Department of Herbology, College of Korean Medicine, Wonkwang University, Iksan 54538, Korea
* Correspondence: rasfin@wku.ac.kr; Tel.: +82-63-850-6985
† These authors contributed equally to this work.

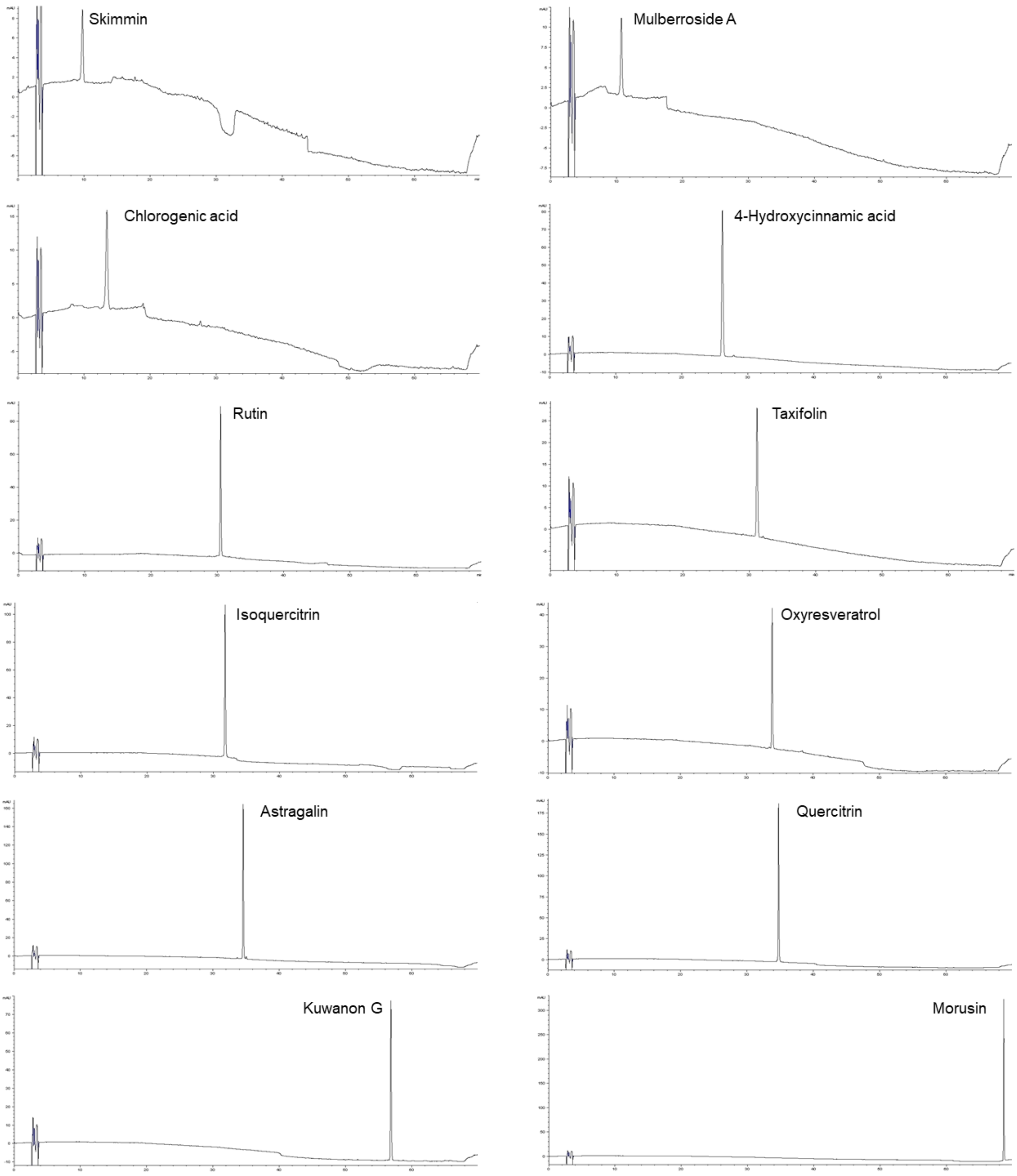

Figure S1. Chromatograms of the 12 marker compounds at the detection wavelength of UV 265 nm.

Table S1. Intra- and interday precisions of the marker compounds.

Compound	Intraday (n = 3)				Interday (n = 3)			
	Initial Conc. (μg/mL)	Detected Conc. (μg/mL)	RSD (%)	Accuracy (%)	Initial Conc. (μg/mL)	Detected Conc. (μg/mL)	RSD (%)	Accuracy (%)
Skimmin	15.00	15.18	0.33	101.21	15.00	15.33	1.75	102.22
	3.75	3.75	2.34	100.13	3.75	3.62	8.69	96.63
	1.88	1.70	1.73	90.46	1.88	1.69	2.16	90.24
Mulberroside A	300.00	310.29	2.24	103.43	300.00	310.41	2.25	103.47
	75.00	78.49	2.23	104.65	75.00	77.13	2.73	102.84
	18.75	18.64	1.31	99.42	18.75	18.56	2.03	99.00
Chlorogenic acid	350.00	350.10	3.31	100.03	350.00	353.63	2.61	101.04
	87.50	86.85	3.11	99.26	87.50	85.18	2.46	97.35
	21.88	20.40	2.74	93.25	21.88	20.03	2.57	91.55
4-Hydroxycinnamic acid	5.00	5.08	2.25	101.57	5.00	5.09	2.14	101.77
	1.25	1.27	2.95	101.89	1.25	1.26	0.54	100.46
	0.31	0.30	4.91	97.30	0.31	0.30	4.64	96.18
Rutin	140.00	144.09	0.36	102.92	140.00	144.53	0.38	103.24
	35.00	34.82	0.73	99.50	35.00	34.63	0.74	98.95
	8.75	7.83	1.04	89.49	8.75	7.68	2.44	87.74
Taxifolin	10.00	10.11	0.66	101.14	10.00	10.16	0.24	101.64
	2.50	2.49	1.59	99.62	2.50	2.54	2.79	101.68
	0.63	0.58	3.09	92.81	0.63	0.57	1.74	90.58
Isoquercitrin	75.00	74.95	1.06	99.94	75.00	75.33	0.96	100.45
	18.75	19.01	1.24	101.41	18.75	18.94	0.89	101.02
	4.69	4.85	1.76	103.37	4.69	4.86	1.77	103.67
Oxyresveratrol	100.00	100.79	1.45	100.79	100.00	101.08	1.28	101.08
	25.00	25.26	1.82	101.06	25.00	25.04	0.28	100.15
	6.25	6.27	0.93	100.35	6.25	6.20	1.66	99.26
Astragalin	50.00	50.93	2.17	101.85	50.00	50.87	2.26	101.74
	12.50	12.58	1.97	100.67	12.50	12.50	0.88	99.99
	3.13	3.12	1.64	99.99	3.13	3.10	1.45	99.17
Quercitrin	25.00	25.53	0.83	102.12	25.00	25.54	0.79	102.15
	6.25	6.28	2.52	100.50	6.25	6.28	2.49	100.48
	1.56	1.46	1.23	93.62	1.56	1.46	1.20	93.26
Kuwanon G	100.00	102.69	0.89	102.69	100.00	102.80	0.95	102.80
	25.00	25.56	1.22	102.22	25.00	25.31	1.90	101.24
	6.25	6.19	1.08	98.98	6.25	6.18	1.27	98.84
Morusin	75.00	76.76	0.79	102.35	75.00	76.87	0.90	102.49
	18.75	19.18	0.92	102.29	18.75	19.02	1.60	101.45
	4.69	4.63	1.30	98.81	4.69	4.61	1.80	98.32

Conc., concentration; RSD, relative standard deviation.

Table S2. Recoveries of the marker compounds (n = 3).

Compound	Initial Conc. (μg/mL)	Spiked Conc. (μg/mL)	Detected Conc. (μg/mL)	Recovery (%)	RSD (%)
Skimmin	3.41	4.00	7.85	110.93	4.31
		2.00	5.52	105.34	6.82
		1.00	4.45	104.08	5.06
Mulberroside A	20.44	30.00	55.03	115.30	3.27
		15.00	36.30	105.71	3.25
		7.500	28.01	100.93	2.07
Chlorogenic acid	86.43	100.00	191.25	104.82	2.10
		50.00	137.27	101.67	6.01
		25.00	113.90	109.89	8.58
4-Hydroxycinnamic acid	0.54	1.00	1.48	94.11	4.35
		0.50	1.03	96.66	3.95
		0.25	0.79	97.51	2.86
Rutin	24.35	30.00	55.92	105.24	0.47
		15.00	39.76	102.72	5.16
		7.50	31.77	98.93	3.82
Taxifolin	1.00	1.00	1.95	95.84	3.91
		0.50	1.46	92.84	3.55
		0.25	1.24	98.40	7.18
Isoquercitrin	17.42	20.00	36.55	95.65	8.09
		10.00	27.27	98.42	5.46
		5.00	22.42	99.95	1.01
Oxyresveratrol	30.47	22.50	49.38	84.06	2.66
		11.25	40.79	91.70	4.54
		5.63	35.69	92.78	2.49
Astragalin	8.29	10.00	19.52	112.28	3.31
		5.00	13.98	113.79	6.98
		2.50	10.97	107.14	7.70
Quercitrin	4.79	6.00	10.24	90.78	6.77
		3.00	7.63	94.59	3.64
		1.50	6.27	98.61	4.29
Kuwanon G	62.36	40.00	101.09	96.82	2.48
		20.00	82.85	102.44	6.78
		10.00	72.48	101.21	1.14
Morusin	57.11	30.00	87.99	102.91	1.86
		15.00	74.04	112.82	2.38
		7.500	65.45	111.16	3.29

Conc., concentration; RSD, relative standard deviation.

Table S3. Mean contents (mg/g) of the marker compounds in the extracts of the *Morus* samples.

Sample	Content (mg/g)[a]											
	**Skimmin**	**Mulberroside A**	**Chlorogenic acid**	**4-Hydroxycinnamic acid**	**Rutin**	**Taxifolin**	**Isoquercitrin**	**Oxyresveratrol**	**Astragalin**	**Quercitrin**	**Kuwanon G**	**Morusin**
SB1	-	146.51 ± 2.74	1.94 ± 0.01	-	-	-	-	0.64 ± 0.02	-	-	24.57 ± 0.22	6.12 ± 0.03
SB2	-	-	0.77 ± 0.04	-	-	-	-	0.71 ± 0.02	-	-	1.29 ± 0.01	5.95 ± 0.02
SB3	-	96.62 ± 2.82	-	-	-	-	-	2.35 ± 0.03	-	-	46.54 ± 0.20	23.80 ± 0.15
SB4	-	0.31 ± 0.05	5.35 ± 0.11	-	-	0.05 ± 0.06	-	0.54 ± 0.02	-	-	0.87 ± 0.03	1.79 ± 0.01
SB5	-	53.65 ± 1.26	2.33 ± 0.04	-	-	0.94 ± 0.02	-	2.81 ± 0.05	-	-	8.97 ± 0.05	5.33 ± 0.00
SB6	-	98.59 ± 2.04	1.72 ± 0.03	-	-	-	-	0.21 ± 0.01	-	-	39.85 ± 0.37	11.37 ± 0.10
SB7	-	-	4.37 ± 0.05	-	-	0.54 ± 0.01	-	1.21 ± 0.04	-	-	0.96 ± 0.15	0.82 ± 0.00
SB8	-	98.69 ± 0.30	1.90 ± 0.02	-	-	-	-	0.57 ± 0.01	-	-	26.24 ± 0.13	7.57 ± 6.58
SB9	-	135.58 ± 2.02	2.60 ± 0.02	-	-	-	-	0.82 ± 0.02	-	-	33.56 ± 0.25	8.96 ± 0.04
SB10	-	4.47 ± 0.06	4.41 ± 0.03	-	-	-	-	0.64 ± 0.02	-	-	32.03 ± 0.15	10.53 ± 0.06
SB11	-	-	28.27 ± 0.32	-	-	0.53 ± 0.01	-	2.53 ± 0.05	-	-	0.91 ± 0.01	3.68 ± 0.01
SB12	-	81.39 ± 0.45	3.45 ± 0.06	-	-	0.08 ± 0.01	-	4.55 ± 0.11	-	-	11.01 ± 0.11	10.99 ± 0.05
SB13	-	160.18 ± 2.37	24.07 ± 0.21	-	-	-	-	0.72 ± 0.03	-	-	52.53 ± 0.18	32.47 ± 0.08
SB14	-	226.07 ± 2.30	-	-	-	-	-	0.46 ± 0.01	-	-	81.05 ± 0.40	34.91 ± 0.19
SB15	-	-	< LOQ	-	-	-	-	0.28 ± 0.01	-	-	0.40 ± 0.02	0.37 ± 0.01
**Mean**	**-**	**100.19 ± 63.62**	**6.77 ± 8.81**	**-**	**-**	**0.43 ± 0.33**	**-**	**1.27 ± 1.19**	**-**	**-**	**24.05 ± 23.17**	**10.98 ± 10.49**
SJ01	-	55.67 ± 1.22	-	0.16 ± 0.00	-	-	-	0.35 ± 0.02	-	-	1.22 ± 0.02	1.05 ±0.00
SJ02	-	110.82 ± 0.52	-	0.31 ± 0.01	-	-	-	17.18 ± 0.36	-	-	5.92 ± 0.04	3.77 ± 0.03
SJ03	-	19.45 ± 0.40	-	0.49 ± 0.01	-	-	-	1.69 ± 0.06	-	-	-	0.81 ± 0.02
SJ04	-	87.05 ± 1.15	-	0.20 ± 0.01	-	-	-	4.72 ± 0.13	-	-	5.79 ± 0.09	4.38 ± 0.01
SJ05	-	4.91 ± 0.06	-	0.27 ± 0.00	-	-	-	0.51 ± 0.02	-	-	2.08 ± 0.08	1.95 ± 0.01
SJ06	-	52.07 ± 0.55	-	0.28 ± 0.00	-	-	-	0.61 ± 0.03	-	-	8.08 ± 0.03	7.33 ± 0.03
SJ07	-	12.40 ± 0.14	-	0.28 ± 0.00	-	-	-	0.60 ± 0.02	-	-	6.95 ± 0.16	3.20 ± 0.02
SJ08	-	76.45 ± 1.43	-	0.27 ± 0.01	-	-	-	0.35 ± 0.01	-	-	0.81 ± 0.01	1.09 ± 0.01
SJ09	-	2.14 ± 0.06	-	0.10 ± 0.00	-	-	-	9.85 ± 0.16	-	-	1.42 ± 0.02	1.18 ± 0.01
SJ10	-	51.08 ± 0.23	-	0.39 ± 0.02	-	-	-	27.75 ± 0.39	-	-	4.39 ± 0.08	3.57 ± 0.03
SJ11	-	18.06 ± 0.23	-	0.08 ± 0.00	-	-	-	4.02 ± 0.06	-	-	1.94 ± 0.05	0.54 ± 0.00
**Mean**	**-**	**44.55 ± 34.61**	**-**	**0.26 ± 0.11**	**-**	**-**	**-**	**6.15 ± 8.47**	**-**	**-**	**3.86 ± 2.54**	**2.63 ± 1.97**
SS01	-	-	3.81 ± 0.05	-	1.10 ± 0.03	-	1.65 ± 0.01	-	-	-	-	0.26 ± 0.00
SS02	-	-	6.04 ± 0.05	0.03 ± 0.00	1.85 ± 0.03	-	1.92 ± 0.02	-	-	-	-	0.08 ± 0.00
SS03	-	-	0.92 ± 0.01	-	0.97 ± 0.01	-	0.29 ± 0.01	-	-	-	-	< LOQ
SS04	-	-	-	-	< LOQ	-	0.05 ± 0.00	-	-	-	-	0.01 ± 0.00
SS05	-	-	0.32 ± 0.00	-	0.11 ± 0.00	-	0.18 ± 0.00	-	-	-	-	< LOQ
SS06	-	-	5.05 ± 0.05	0.03 ± 0.00	3.28 ± 0.04	0.07 ± 0.00	1.05 ± 0.01	-	-	-	-	0.01 ± 0.00
SS07	-	-	0.55 ± 0.00	0.03 ± 0.00	0.46 ± 0.01	0.02 ± 0.00	0.13 ± 0.00	-	-	-	-	-
SS08	-	-	7.02 ± 0.02	0.01 ± 0.00	2.26 ± 0.01	0.05 ± 0.00	0.71 ± 0.00	-	-	-	-	0.22 ± 0.39
SS09	-	-	1.15 ± 0.01	0.02 ± 0.00	1.14 ± 0.01	0.03 ± 0.00	0.24 ± 0.00	-	-	-	-	< LOQ

SS10	-	-	6.33 ± 0.03	0.05 ± 0.00	1.72 ± 0.01	0.07 ± 0.00	0.42 ± 0.01	-	-	-	-	< LOQ
SS11	-	-	2.00 ± 0.01	0.04 ± 0.00	1.01 ± 0.01	0.02 ± 0.00	0.23 ± 0.00	-	-	-	-	< LOQ
SS12	-	-	2.11 ± 0.01	-	0.94 ± 0.02	-	1.03 ± 0.00	-	-	-	-	0.10 ± 0.00
**Mean**	**-**	**-**	**3.21 ± 2.40**	**0.03 ± 0.01**	**1.35 ± 0.84**	**0.04 ± 0.02**	**0.66 ± 0.60**	**-**	**-**	**-**	**-**	**0.11 ± 0.10**
SY01	0.19 ± 0.00	-	14.59 ± 0.01	-	2.76 ± 0.02	-	1.29 ± 0.00	-	0.48 ± 0.00	-	-	-
SY02	0.54 ± 0.04	-	19.68 ± 0.03	-	2.24 ± 0.01	-	4.15 ± 0.09	-	1.37 ± 0.02	-	-	-
SY03	0.09 ± 0.01	-	21.92 ± 0.02	-	4.57 ± 0.04	-	8.29 ± 0.07	-	2.81 ± 0.03	-	-	-
SY04	0.13 ± 0.00	-	19.15 ± 0.02	-	5.23 ± 0.09	-	7.76 ± 0.03	-	2.40 ± 0.01	-	-	-
SY05	0.03 ± 0.01	-	14.88 ± 0.03	-	2.89 ± 0.01	-	4.63 ± 0.05	-	1.76 ± 0.02	-	-	-
SY06	0.11 ± 0.01	-	10.86 ± 0.01	-	2.31 ± 0.01	-	4.85 ± 0.06	-	1.75 ± 0.00	-	-	-
SY07	-	-	0.01 ± 0.04	-	< LOQ	-	2.21 ± 0.05	-	0.96 ± 0.02	-	-	-
SY08	0.40 ± 0.01	-	26.97 ± 0.07	-	1.88 ± 0.01	-	5.39 ± 0.07	-	2.58 ± 0.05	-	-	-
SY09	0.11 ± 0.02	-	9.39 ± 0.11	-	2.37 ± 0.04	-	3.58 ± 0.03	-	1.64 ± 0.02	-	-	-
SY10	0.20 ± 0.01	-	25.63 ± 0.09	-	3.84 ± 0.04	-	8.78 ± 0.09	-	3.75 ± 0.01	-	-	-
SY11	0.62 ± 0.05	-	20.33 ± 0.01	-	4.30 ± 0.03	-	4.16 ± 0.10	-	2.05 ± 0.01	-	-	-
SY12	1.18 ± 0.03	-	38.97 ± 0.05	-	5.46 ± 0.01	-	2.47 ± 0.14	-	1.17 ± 0.10	0.41 ± 0.03	-	-
SY13	0.45 ± 0.01	-	19.85 ± 0.16	-	3.62 ± 0.04	-	1.16 ± 0.10	-	0.55 ± 0.04	0.15 ± 0.00	-	-
SY14	0.42 ± 0.02	-	33.76 ± 0.27	-	3.93 ± 0.09	-	1.76 ± 0.19	-	0.81 ± 0.05	0.38 ± 0.02	-	-
SY15	0.29 ± 0.02	-	25.70 ± 0.14	-	5.13 ± 0.08	-	2.88 ± 0.16	-	1.06 ± 0.05	0.31 ± 0.02	-	-
SY16	0.54 ± 0.01	-	27.71 ± 0.18	-	4.67 ± 0.03	-	5.31 ± 0.27	-	2.60 ± 0.07	-	-	-
SY17	1.40 ± 0.01	-	20.12 ± 0.20	-	0.78 ± 0.01	-	1.25 ± 0.08	-	0.54 ± 0.03	0.06 ± 0.01	-	-
SY18	1.20 ± 0.04	-	4.16 ± 0.01	-	0.28 ± 0.01	-	0.21 ± 0.00	-	0.11 ± 0.01	-	-	-
SY19	0.97 ± 0.02	-	26.06 ± 0.13	-	2.26 ± 0.07	-	2.59 ± 0.03	-	1.05 ± 0.01	-	-	-
**Mean**	**0.49 ± 0.41**	**-**	**19.99 ± 9.36**	**-**	**3.25 ± 1.47**	**-**	**3.83 ± 2.42**	**-**	**1.55 ± 0.93**	**0.26 ± 0.14**	**-**	**-**

[a] Contents (mg/g) are represented as 'mean value ± standard deviation'; '-', not detected; < LOQ, under the limit of quantification; SB, root barks; SJ, twigs; SS, fruits; SY, leaves.

Table S4. The samples of different medicinal parts of *Morus alba* L.

Code No.	Herbal name	Pharmaceutical name	Medicinal part	Location of collection
SB01	Sang-Baek-Pi	Mori Radicis Cortex	Root barks	Samcheok, Gangwon, Korea
SB02	Sang-Baek-Pi	Mori Radicis Cortex	Root barks	-
SB03	Sang-Baek-Pi	Mori Radicis Cortex	Root barks	-
SB04	Sang-Baek-Pi	Mori Radicis Cortex	Root barks	-
SB05	Sang-Baek-Pi	Mori Radicis Cortex	Root barks	Sichuan, China
SB06	Sang-Baek-Pi	Mori Radicis Cortex	Root barks	Yeongcheon, Gyeongbuk, Korea
SB07	Sang-Baek-Pi	Mori Radicis Cortex	Root barks	China
SB08	Sang-Baek-Pi	Mori Radicis Cortex	Root barks	Yeongcheon, Gyeongbuk, Korea
SB09	Sang-Baek-Pi	Mori Radicis Cortex	Root barks	-
SB10	Sang-Baek-Pi	Mori Radicis Cortex	Root barks	-
SB11	Sang-Baek-Pi	Mori Radicis Cortex	Root barks	-
SB12	Sang-Baek-Pi	Mori Radicis Cortex	Root barks	Wanju, Jeonbuk, Korea
SB13	Sang-Baek-Pi	Mori Radicis Cortex	Root barks	Dangjin, Chungnam, Korea
SB14	Sang-Baek-Pi	Mori Radicis Cortex	Root barks	Taean, Chungnam, Korea
SB15	Sang-Baek-Pi	Mori Radicis Cortex	Root barks	China
SJ01	Sang-Ji	Mori Ramulus	Twigs	Samcheok, Gangwon, Korea
SJ02	Sang-Ji	Mori Ramulus	Twigs	Sichuan, China
SJ03	Sang-Ji	Mori Ramulus	Twigs	Yeongcheon, Gyeongbuk, Korea
SJ04	Sang-Ji	Mori Ramulus	Twigs	-
SJ05	Sang-Ji	Mori Ramulus	Twigs	Gosan, Wanju, Korea
SJ06	Sang-Ji	Mori Ramulus	Twigs	Taean, Chungnam, Korea
SJ07	Sang-Ji	Mori Ramulus	Twigs	Dangjin, Chungnam, Korea
SJ08	Sang-Ji	Mori Ramulus	Twigs	Korea
SJ09	Sang-Ji	Mori Ramulus	Twigs	Busan, Korea
SJ10	Sang-Ji	Mori Ramulus	Twigs	Busan, Korea
SJ11	Sang-Ji	Mori Ramulus	Twigs	Busan, Korea
SS01	Sang-Sim-Ja	Mori Fructus	Fruits (immature)	-
SS02	Sang-Sim-Ja	Mori Fructus	Fruits (immature)	-
SS03	Sang-Sim-Ja	Mori Fructus	Fruits (immature)	Sichuan, China
SS04	Sang-Sim-Ja	Mori Fructus	Fruits (immature)	China
SS05	Sang-Sim-Ja	Mori Fructus	Fruits (immature)	China
SS06	Sang-Sim-Ja	Mori Fructus	Fruits (immature)	Wanju, Jeonbuk, Korea
SS07	Sang-Sim-Ja	Mori Fructus	Fruits (mature)	Wanju, Jeonbuk, Korea
SS08	Sang-Sim-Ja	Mori Fructus	Fruits (immature)	Taean, Chungnam, Korea
SS09	Sang-Sim-Ja	Mori Fructus	Fruits (mature)	Taean, Chungnam, Korea
SS10	Sang-Sim-Ja	Mori Fructus	Fruits (immature)	Dangjin, Chungnam, Korea
SS11	Sang-Sim-Ja	Mori Fructus	Fruits (mature)	Dangjin, Chungnam, Korea
SS12	Sang-Sim-Ja	Mori Fructus	Fruits (immature)	China
SY01	Sang-Yeop	Mori Folium	Leaves	Samcheok, Gangwon, Korea
SY02	Sang-Yeop	Mori Folium	Leaves	Samcheok, Gangwon, Korea
SY03	Sang-Yeop	Mori Folium	Leaves	Jangsu- Jeonbuk, Korea
SY04	Sang-Yeop	Mori Folium	Leaves	Jangsu- Jeonbuk, Korea
SY05	Sang-Yeop	Mori Folium	Leaves	Jangsu- Jeonbuk, Korea
SY06	Sang-Yeop	Mori Folium	Leaves	Jangsu- Jeonbuk, Korea
SY07	Sang-Yeop	Mori Folium	Leaves	-
SY08	Sang-Yeop	Mori Folium	Leaves	Geochang- Gyeongnam, Korea
SY09	Sang-Yeop	Mori Folium	Leaves	Sichuan, China
SY10	Sang-Yeop	Mori Folium	Leaves	Yeongcheon, Gyeongbuk, Korea
SY11	Sang-Yeop	Mori Folium	Leaves	-
SY12	Sang-Yeop	Mori Folium	Leaves	Wanju, Jeonbuk, Korea
SY13	Sang-Yeop	Mori Folium	Leaves	Dangjin, Chungnam, Korea
SY14	Sang-Yeop	Mori Folium	Leaves	Taean, Chungnam, Korea
SY15	Sang-Yeop	Mori Folium	Leaves	Busan, Korea
SY16	Sang-Yeop	Mori Folium	Leaves	Korea
SY17	Sang-Yeop	Mori Folium	Leaves	Busan, Korea
SY18	Sang-Yeop	Mori Folium	Leaves	Busan, Korea
SY19	Sang-Yeop	Mori Folium	Leaves	Busan, Korea

'-', unclear location.
